# From self-harm to shock: A case report of mercury poisoning and cutaneous injury

**DOI:** 10.1016/j.jdcr.2025.10.006

**Published:** 2025-10-23

**Authors:** Juliana M. O’Reilly, Yizhen E. Martin, Mary Beth Gadarowski, Christian Lamb, Joseph Maddry, William Schaffenburg

**Affiliations:** a Graduate Medical Education, Walter Reed National Military Medical Center, Bethesda, Maryland; b Department of Dermatology, Mike O'Callaghan Military Medical Center, Las Vegas, Nevada; c Department of Dermatology, Keesler Medical Center, Biloxi, Mississippi; d Department of Medicine, 88th Medical Group, Wright Patterson Air Force Base, Ohio; e Department of Emergency Medicine and Toxicology, San Antonio Uniformed Services Health Education Consortium, San Antonio, Texas; f Department of Dermatology, San Antonio Uniformed Services Health Education Consortium, San Antonio, Texas

**Keywords:** mercuric chloride, mercury poisoning, morbilliform rash, self-harm, toxic shock, ulcer

## Introduction

Mercuric chloride is an inorganic compound that is highly toxic.^1^ The intravenous and subcutaneous injection of mercuric chloride is a rare method of self-harm. Few cases have documented the injection and oral ingestion of various mercury compounds.^1^, ^2^, ^3^, ^4^

Mercury poisoning is not a typical exposure history that clinicians encounter, which can make diagnosis challenging, particularly if a patient is not forthcoming about their history. Herein, we describe a case of mercury poisoning as a means of self-harm masquerading as toxic shock syndrome.

## Case report

**Fig 1:**
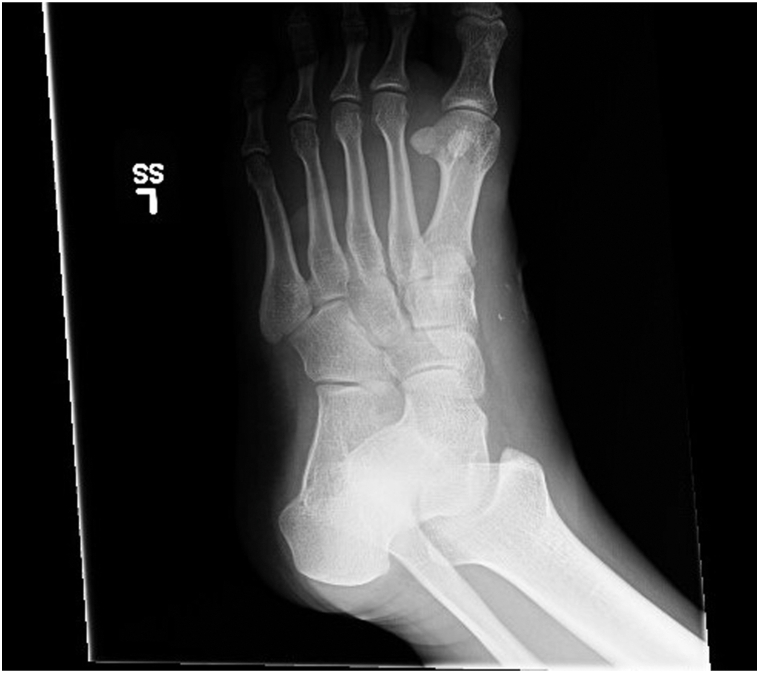
Left foot radiograph demonstrated focal radiopaque particulate material at mid-dorsal foot.

We present a 22-year-old male with alcohol abuse, self-injurious behaviors, prior suicide attempts, and major depressive disorder on mirtazapine. He presented with diffuse erythema, swelling, and blistering of the left foot and ankle, initially suspected as an ankle fracture, along with an ulcer in the antecubital fossa. He reported subjective fever and cough. Exam showed diffuse morbilliform rash, pitting edema to the knee and bullae on the left foot. Imaging excluded abscess, necrotizing fasciitis, or fracture but revealed sand-like particles in the soft tissues (Fig 1). Laboratory results showed monocytosis, eosinophilia, lymphopenia, and acute kidney injury (AKI) (creatinine 1.2 from baseline 0.8-0.9 mg/dL). During the evaluation, he developed septic shock requiring intravenous fluids and vasopressors.

**Fig 2:**
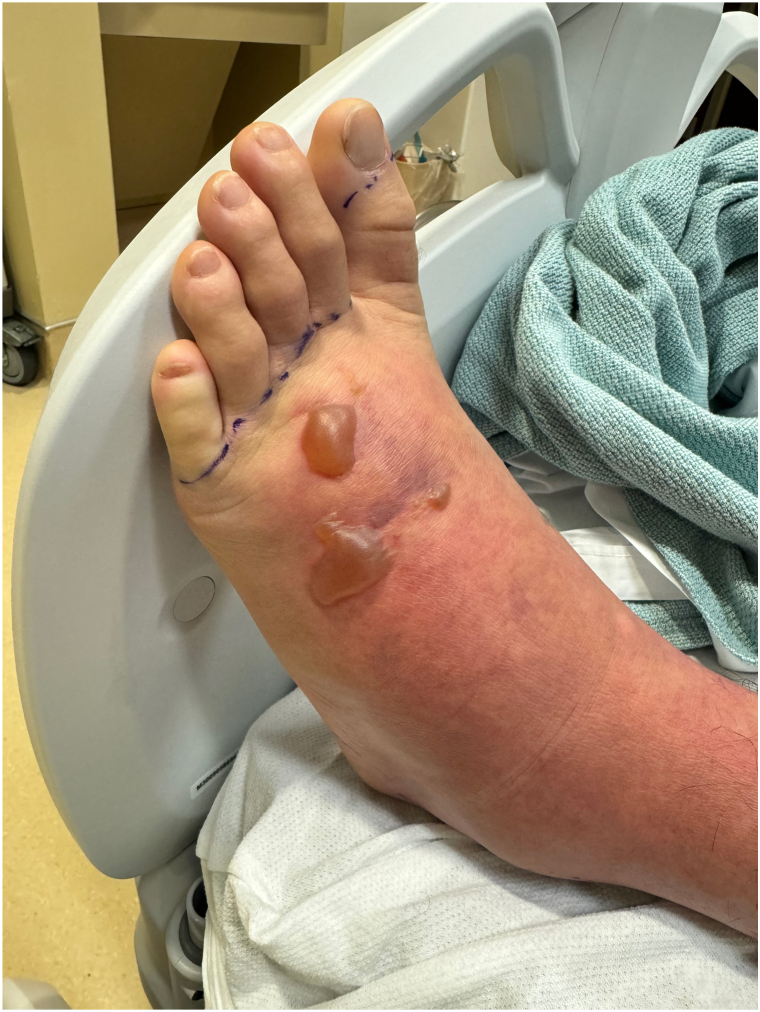
Two tense bullae with background blanchable erythema and edema of left dorsal foot.

**Fig 3:**
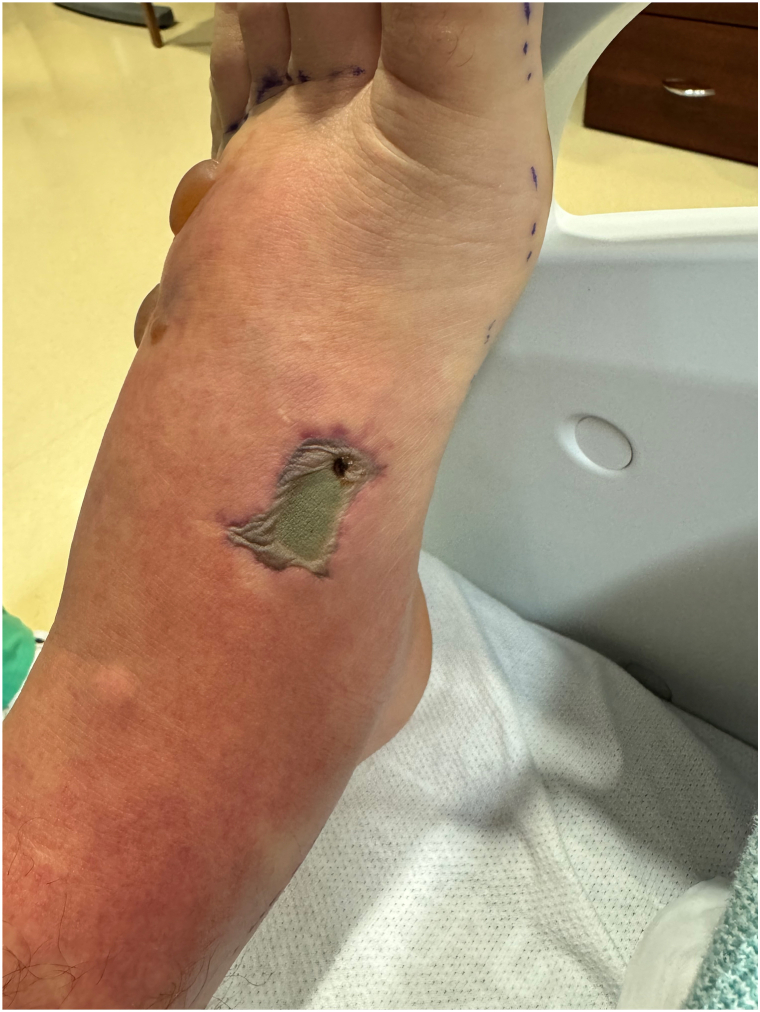
Sharply angulated, *rectangular* necrotic ulcer of left medial dorsal foot.

Dermatology was consulted due to concerns for toxic shock syndrome in the setting of trauma to the left foot. Examination revealed a diffuse morbilliform rash sparing the hands and feet. The patient was warm to the touch, with blanching erythema, and had shotty inguinal lymphadenopathy. The dorsal foot was edematous with 2 tense bullae. A well-circumscribed, rectangular necrotic-appearing ulcer with gray adherent material was present on the medial aspect of the dorsal foot (Figs 2 and 3). Due to concerns for delayed wound healing, particularly given the anatomical site and degree of edema, a skin biopsy was deferred. Wound cultures from the foot and antecubital fossa and blood cultures were negative. While the patient did not have typical signs of toxic shock syndrome (such as sandpaper texture, mucosal involvement or acral desquamation), an immune response secondary to infectious etiology was considered.

During his hospital course, the patient eventually disclosed he procured mercuric chloride online and injected it into his foot and arm as a means of self-harm. Given this information and his AKI, toxicology was subsequently consulted for potential mercury poisoning, and an elevated urinary mercury level >100 μg/L (normal reference range: 0-20 μg/L) confirmed his toxic exposure. Supportive wound care led to uncomplicated healing, and the patient was discharged with on-going psychiatric care without any dermatologic sequelae.

## Discussion

Mercury toxicity presents with varied manifestations depending on the form and duration of exposure. Mercury exists in elemental, inorganic, and organic forms and is mainly used in industrial applications such as wood preservation, embalming, disinfectants, photographic intensifiers, and leather tanning. Environmental exposures, including skin-lightening creams, older toys, and some tattoo pigments, may also cause systemic or cutaneous findings similar to intentional injections.^5^ Acute and chronic exposure may lead to systemic toxicity.

Acute exposure may present with mucosal changes, gastrointestinal symptoms, fluid imbalance, gingival irritation, and renal tubular injury.^5^ Chronic exposure primarily impacts the central nervous system and renal function, often causing tremors, behavioral changes such as irritability and social withdrawal, and, in some cases, nephrotic syndrome.^5^

Lavery et al described the distinct cutaneous syndromes associated with mercury poisoning as acrodynia (pink disease), acute generalized exanthematous pustulosis, symmetrical drug-related intertriginous and flexural exanthema, mercury exanthema, contact dermatitis, hyperpigmentation and cutaneous granuloma.^6^^,^^7^ The cutaneous features of elemental mercury exposure are erythematous papular eruption, lichenoid reaction, and blue line across gingiva.^7^ Inorganic mercury exposure may present as acrodynia, blue line across gingiva and tongue, stomatitis, and slate-gray pigmentation (exogenous ochronosis).^7^ Subcutaneous injections of mercury have produced acute reactions of local pain, burning, swelling, erythema, blistering, necrosis, acute inflammatory and sterile abscess production.^8^ Late reactions to subcutaneous injections include foreign body giant cell reaction, granulomas, fibrosis, membranous fat necrosis, and local lymphadenopathy.^8^ In the setting of known subcutaneous mercury injections, surgical debridement and serial mercury level monitoring may be indicated.^2^

Management of mercury toxicity focuses on preventing exposure and providing supportive care.

While no specific antidote exists, chelation may be used in symptomatic patients or those with high mercury levels. Common chelators include dimercaprol (BAL), D-penicillamine, DMPS, and succimer (DMSA), though their use is limited by binding of essential metals. Akaras et al recently suggested that carvacrol, a monoterpenoid phenol, may offer neuroprotective effects in mercuric chloride–induced neurotoxicity.^9^

In this case, mercury exposure was only recognized after the patient disclosed the full history. As the patient improved with supportive care, biopsy was not pursued, and the lack of histologic confirmation remains a limitation. Characteristic histologic features of elemental mercury injection is the presence of circular mercury deposits within the tissue, often surrounded by an artificial retraction space from tissue processing.^10^ We encourage providers in similar clinical scenarios to consider a biopsy, as delayed reactions such as foreign body giant cell response, granulomas, fibrosis, and membranous fat necrosis may occur. Long-term dermatologic follow-up is recommended to monitor for late sequelae.

This case illustrates how an unusual presentation, such as morbilliform rash, geometric ulcerations, and significant asymmetric edema of the lower extremity, raised concerns beyond the patient's initial history. The patient’s initial presentation with AKI, followed by an elevated urine mercury level, ultimately confirmed the diagnosis of mercury poisoning. This case highlights the broad spectrum of clinical presentations following mercury injection, contributes to the literature through its unique features, and emphasizes the need for dermatologist vigilance in recognizing such manifestations.

## Conflicts of interest

None disclosed.
